# Post-GWAS knowledge gap: the how, where, and when

**DOI:** 10.1038/s41531-020-00125-y

**Published:** 2020-09-09

**Authors:** Steven E. Pierce, Alix Booms, Jordan Prahl, Edwin J. C. van der Schans, Trevor Tyson, Gerhard A. Coetzee

**Affiliations:** Center for Neurodegenerative Science, Van Andel Institute, Grand Rapids, MI USA

**Keywords:** Genomics, Neurological disorders

## Abstract

Genetic risk for complex diseases very rarely reflects only Mendelian-inherited phenotypes where single-gene mutations can be followed in families by linkage analysis. More commonly, a large set of low-penetrance, small effect-size variants combine to confer risk; they are normally revealed in genome-wide association studies (GWAS), which compare large population groups. Whereas Mendelian inheritance points toward disease mechanisms arising from the mutated genes, in the case of GWAS signals, the effector proteins and even general risk mechanism are mostly unknown. Instead, the utility of GWAS currently lies primarily in predictive and diagnostic information. Although an amazing body of GWAS-based knowledge now exists, we advocate for more funding towards the exploration of the fundamental biology in post-GWAS studies; this research will bring us closer to causality and risk gene identification. Using Parkinson’s Disease as an example, we ask, how, where, and when do risk loci contribute to disease?

Genome-wide association studies (GWAS), and newer whole-genome sequencing (WGS) studies, are the most recent in a series of strategies to determine the genetic underpinning of complex human diseases^1^. GWAS evolved from hypothesis-driven candidate gene association studies (CGAS) during the last quarter of the 20th century. As technology developed, unbiased, genome-wide searches became possible. Nevertheless, like CGAS, these were originally intended to produce two types of valuable knowledge. Firstly, researchers hoped to uncover the underlying molecular mechanisms by which a disease originates, and in particular, identify all relevant genes and gene variants (i.e., disease causality). Secondly, genetic associations were expected to provide useful diagnostic markers for both public and individual health decisions. Unfortunately, it has turned out to be harder than expected to make the leap from statistical risk associations to mechanistic insight, and very few experiments are done based on GWAS results^2^. Instead, emphasis (or at least progress) has shifted to favor the second objective. Polygenic risk scores are being increasingly refined to give higher confidence predictions of disease risk for people of increasingly diverse populations. The utility of this has been reviewed^3,4^. For some diseases, including diabetes, breast cancer, and coronary heart disease, polygenic risk informs treatment options and helps to stratify disease subtypes. However, in the case of Parkinson’s Disease (PD) and similar diseases (Multiple System Atrophy, Dementia with Lewy bodies, Progressive supranuclear palsy, for example), our understanding of the disease is too limited, and treatment options too few, for polygenic scoring to currently have practical use. There is no treatment that alters disease course and management for the related diseases is essentially identical. Hence, for PD, predictive genetic diagnostics will remain largely irrelevant to patients without other major advancements. These include more treatment targets and a more detailed understanding of all of the many relevant biological pathways underpinning pathogenesis. Notably, these topics closely match the predicted but unrealized value in GWAS^5^. But, as several recent comment papers have suggested or implied, for the PD field to make headway it must reorient its conceptualization of existing GWAS results and funding agencies should place greater emphasis on post-GWAS research^6–10^. Here, we argue that, in the PD field, the potential for, and difficulty in acquiring, GWAS-based disease insight is underappreciated by those who are not themselves engaged in post-GWAS research; this is partially due to underinvestment in mechanistic follow-up studies and the resulting dearth in testable hypothesis.

There is a small set of laudatory examples showing the potential for uncovering new biology and targets based on GWAS results. A commonly cited example is that of obesity and in the role of intronic variants altering the regulation of *FTO1* and other nearby gene expression levels in the hypothalamus, which then affects feeding behavior^11^. Unfortunately, in the case of PD, very few post-GWAS PD studies even exist. This makes the potential utility difficult to convey to PD researchers. The most prominent was published in 2016^12^, and showed that, in neuronal iPSCs, a *SNCA* intronic enhancer overlapped PD associated variants. At one single nucleotide polymorphisms (SNPs), they confirmed allele-dependent transcription factor binding and, using careful genetic manipulation, detailed allele-dependent expression of *SNCA*. However, this study did not report the variants’ effects on global gene expression, nor on cell morphology. Moreover, the functional variant identified accounted for only a small portion of the statistical risk signal (at that risk locus) and other functional variants were not identified. In short, a large amount of work lead to this paper which narrowly described a possible risk mechanism and confirmed a possible cell type but was not intended to address PD pathogenesis directly. Crucially, since few similar studies are currently published, there seems to be a catch-22 where post-GWAS studies are underfunded because they are quite difficult and lack obvious payoff in terms of immediate disease relevance. Unfortunately, disease insight would come from a second tier of research that seeks to link risk mechanisms to PD but that requires an ecosystem of fundamental mechanistic studies which doesn’t exist.

The large and unexpected hurdle in conducting post-GWAS studies is due to complexity and ambiguity of the risk signals. Instead of identifying protein-altering variants, ~90% of all GWAS risk variants lie in non-coding DNA^8,13^. In most cases these probably alter the sequence at regulatory elements (RE) such as enhancers, promoters, various non-coding RNAs, and CTCF binding sites; and may also alter other elements including splice junctions. Risk variants thereby change the regulation of gene expression (relative to the alternative allele) in a cell-specific manner, in concordance with RE activity^14^. Enhancers are most numerous and contain a large proportion of risk variants. These are short sequences of DNA which bind activator or repressor proteins and regulate the transcription of one or more target genes through variable proximity to promoters. However, for enhancers, and to a lesser extent for other REs, the target genes are not immediately obvious. A possibly greater problem is due to the density of variants (most of which are SNPs) in the human genome. In 2015, the 1000 genomes project reported 84.7 million SNPs with a minor allele frequency of >1% in the human genome^15^. While high SNP density enables GWAS it also complicates the examination of the mechanisms underpinning that risk. Closely spaced SNPs in high linkage disequilibrium (LD) makes identifying functional variants, let alone risk-driving variants, challenging because any one or more could be causal. GWAS risk loci are usually reported via the most significant index SNPs that tags a locus (based on the SNPs used in discovery micro-array chips), but these act as surrogates for dozens to thousands of other significantly associated variants that may span more than a mega-base and overlap multiple regulatory elements. Among the 90 independent PD risk signals, at 78 loci, there are more than 6500 credible risk SNPs or SNPs of interest, i.e., those variants both significant and plausibly contributing to the risk signals based on other criteria^16^. Most of these variants are probably not affecting disease processes, but instead are linked to causal variants through LD; although superfluous variants can also be functional and affect genes unrelated to the original GWAS. This complexity is often treated as noise which can be partially offset by merging multiple data types including Roadmap epigenomic, GTEx eQTL (SNP variant/gene expression association pairs), gene expression, and data from representative chromosomal conformation experiments^17,18^. PD risk gene sets have been thereby defined that contain bona fide PD causal genes and show high levels of informative pathway enrichment^19–23^. However, the precision of these lists is illusory, and for nearly all individual GWAS ‘hits’ there is very high uncertainty, with many false positives and false negatives.

For gene mutations linked to familial PD, there are clear experimental paths forward. Correspondingly, familial risk genes^24^ generate an average of 30 (median: 20) Parkinson’s publications per gene per year (Fig. 1). In contrast, the number of GWAS risk loci seem to multiply every year without resulting in mechanistic experiments. For GWAS there is an observable gap in utilization; GWAS-based PD genes (which have been published for at least five years and which have a PD publication record) average only 1.6 (median: 1.3) publications per year (Fig. 1). Indeed, for the most studied familial gene, PubMed currently (March, 2020) lists ~1800 publications with the words “Parkinson’s” or “neurodegeneration” and “LRRK2” in the title or abstract; almost 5 times as many as for the rest of the 62 non-familial PD GWAS “genes of interest” put together.

**Fig. 1:**
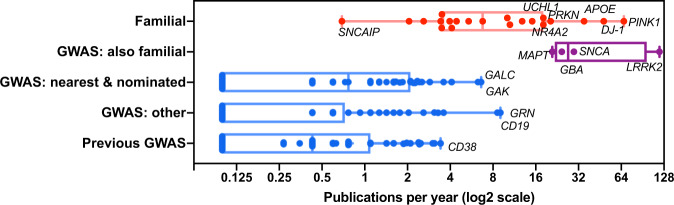
The research gap between familial and GWAS Parkinson’s genes. As a proxy for research effort, PubMed was queried for publications that include the words, “Parkinson’s” or “neurodegeneration” and the standard gene abbreviation (protein and full names were ignored) in the title or abstract. The total number of studies was divided by the number of years since the gene was first formally linked to PD. The most recent PD meta-analysis reported relevant genes based on multiple criteria, those which are both nearest to the risk signal and supported by mendelian randomization. eQTL data are listed as “GWAS nearest & nominated”, the remainder as “GWAS other”. Genes which were reported in the 2012, 2014, or 2017 but not in the 2019 PD meta-analysis are titled “Previous GWAS”. Mean, 25th, and 75th percentiles are shown.

The relatively small effect sizes of GWAS risk is not sufficient to justify the lack of research. A small disease risk does not imply small phenotypic effects nor imply that related pathways could not be exploited for disease modification. However, the set of nominated GWAS driver genes is in flux and researchers may be unconvinced by the complex statistical inferences upon which they are based. Instead for GWAS genes, like familial genes, to lead to clinically relevant research, the underlying risk mechanisms should be verified experimentally. In other words; each risk locus must be shown to function in a specific cell type, at a particular time with respect to the disease, and via a defined allele-dependent mechanism. A basic conceptual benchmark for understanding is knowing how, where, and when each risk variant functions.

## The how: GWAS mechanisms of PD risk

More than 95% of PD GWAS risk variants reside in non-exonic DNA^16^; the effects on protein functions are not currently predictable. Still, a general framework exists wherein risk variants modulate allele-dependent regulatory element function or alter the transcript of non-coding RNA. Since most risk SNPs are found at enhancers the majority of risk mechanisms will be encompassed by matching enhancers with the promoters of the target genes they regulate. Chromatin biofeatures including histone modifications, nucleosome positioning, and transcription factor binding motifs are often used to reduce the number of possible causal SNPs at each locus in a particular cell type^25^. This determines which of several closely linked risk SNPs could be functional (in a context-dependent manner). As causality (with respect to the disease) requires functionality, the first experimental challenge is in identifying the gene or genes that show allele-dependent changes associated with those SNPs. While enhancer-gene interactions are directly related to distance, more than 40% of enhancers skip over the nearest gene^26^ and can even regulate genes on other chromosomes^27^. In the most recent PD GWAS, risk genes were proposed based in part on large scale eQTL studies, which find genes showing allele-dependent expression changes^16,19,28^. However, eQTL analyses often have many false positives^29^ and negatives, due to bias for accessible tissues, cell type heterogeneity in samples, multiple hypotheses penalties, and population bias among donors. A recent review from the Shendure lab^30^ highlights the complexity of enhancer/target-gene matching and lists CRISPR methods as most promising, these are time consuming and can generate off-target effects, which must also be controlled for. These techniques include single cuts to create lesions, dual cuts to remove the entire enhancer, CRISPRi and CRISPRa to inhibit or activate enhancers, respectively without genomic editing. These methods should be matched with chromatin looping assays that identify topological associate domains and more can reveal direct enhancer/promoter interactions which are variable with domains in a tissue-dependent manner^31^. Whereas this will designate putative risk mechanisms based on enhancer and promoters, resolution is often not optimal, and it should be noted that risk arising from other processes, such as regulatory non-coding RNAs will require other approaches.

CRISPR editing to regulatory elements can result in expression changes to either very few or a great many genes, small or large changes in magnitude, and direct or mostly indirect effects. In all cases, researchers should publish their complete results, instead of restricting their data to the gene of interest.

## The where: substantia nigra and glia as sources of PD risk

PD is a neurological disorder characterized by the selective degeneration of dopaminergic neurons within the substantia nigra^32^. Cell types, such as microglia and astrocytes^33^ in the brain may also be involved. Microglia are central players in neuro-inflammation, a process implicated in PD etiology. Postmortem studies have identified activated microglia in key brain regions associated with cell death and pathology^34^. However, dopaminergic neurons remain on the forefront of proposed mechanisms. This is because the loss of dopamine signaling from the substantia nigra to the striatum is the primary cause of the distinctive motor symptoms associated with PD.

There is also emerging evidence that the adaptive immune system plays an active role in the pathogenesis of PD. Several recent studies have shown that T-cells may be involved in regulating the aggregation and propagation of α-syn^35^. These responses of the adaptive immune system may explain the association of HLA alleles seen in GWAS, but whether these are manifested in the MHC-I expressing neurons and/or antigen-presenting glia of the CNS or in the immune cells themselves is unresolved.

Since we don’t know in what tissue or condition risk loci may function, two approaches can be taken.

Firstly, as alluded to above, a locus-centric approach can be used and an unbiased query of tissue- or cell-specific genomic activity can be made. Enrichment of variants in active regions indicates the most appropriate tissue(s) for a given locus^14,36,37^. This approach requires a sufficiently detailed and unbiased encyclopedia of regulatory elements which does not necessarily exist for many tissue types. Alternatively, one can take a best guess based on disease knowledge and choose a likely model system. In this case, the first step is simply to screen out all of the risk loci that appear inert in that model context, by screening for active regulatory elements. Those risk variants that remain can then be examined in detail because each *might* be functional, and this activity *might* be important in disease acquisition or progression (i.e., causal). Ideally, disease-relevant phenotypes will be observable and conditional on genetic variability or manipulation. We recommend, for versatility, choosing models based on induced pluripotent stem cells (iPSCs). The same progenitors can be differentiated into multiple cell types (such as dopaminergic neurons and microglia) and can be followed through time in carefully controlled conditions. They can either be genetically edited at risk variants or derived from different genetic backgrounds to take advantage of natural heterogeneity. Some studies have compared disease vs. healthy patient-derived iPSCs for this purpose^38^.

Some common objections to iPSCs are that they are too simple, and do not replicate the complex heterogeneous environment of an intact organ or body. This can, in principle, be somewhat offset by co-culturing different cell types in 3-D culture systems. In practice, this seems to be rare and would still not account for the milieu of external stimuli, such as infections, metabolic variability, and chemical perturbations encountered in vivo. Another issue is the degree to which iPSC derived models faithfully represent their intended cell type. There is large variation within the iPSC cell population, and, for instance, by single-cell RNA-sequencing, some markers do not correspond to ex vivo tissue comparisons. This argument could suggest the use of some embryonic cell lines more closely differentiated towards the intended cell type. Finally, the process of creating iPSCs removes epigenetic marks, particularly methylation, which may be relevant to risk and disease mechanisms.

## The when: the multiple-hit hypothesis of PD risk

Parkinsonian symptoms most often emerge late in life after a sufficiently severe degradation of the nigrostriatal dopaminergic system has occurred. In this framework, it is useful to distinguish 2 types of risk, which may be identified via GWAS. The first represents the likelihood for an increased toxicity load or insult level falling upon the nigrostriatal systems of the at-risk population. The second represents a decreased capacity or robustness inherent to those nigrostriatal systems. It is important to keep in mind that genetic risk mechanisms can come into play during any stage of an individual’s lifespan, from early development to eventual death. We do not know which mechanism predominates among PD risk loci; and they differ in terms of the expected results following experimental manipulation.

We hypothesize that at least some germline genetic risk loci are involved during developmental phases of dopaminergic differentiation of the substantia nigra^39^. It is well-known that dynamic regulation of gene expression occurs during cellular differentiation. This was recently exemplified in time-series RNA-sequencing data, with over 16 time points during the differentiation of iPSC into cardiomyocytes^40^. Consequently, we propose that some genetic variation at PD risk SNPs impose functional consequences during neuron development, even in utero, and these could also act as a first step of multiple risk hits during a person’s lifetime^41^. For example, people born with fewer dopamine neurons may be more susceptible to the (unknown) events that cause disease later in life. Variability in the number of midbrain dopaminergic neurons is large (4 independent studies all showed dramatic variation in control subjects) and may affect PD, as was recently proposed^42^; this in turn may be influenced by germline variation.

Again, we recommend the use of iPSC dopaminergic neurons, microglia and astrocytes, which can be followed during differentiation in culture. We hypothesize that in individuals who develop PD, germline variation may be involved during early development representing a “first hit”^39^. Consequently, in combination with subsequent “second hits”, they manifest PD symptoms. iPSCs may be optimal for risk which manifests early in development. Risk that requires a cumulative environmental context such as that occurring over decades will be missed. Although, some strategies are being pursued to either induce from old cells or artificially “age” iPSCs^43^.

Nevertheless, identification of mechanisms which either limit the development of dopaminergic neurons in early life or cause these neurons to be more susceptible to injury in later life is crucial for developing potential treatments for PD.

## Conclusion

Recent twin studies have shown that the heritability of PD is 27% overall and can be as high as 83% in monozygotic twins diagnosed before the age of 50^44^. Increasingly large GWAS meta-analysis (for PD: 40,000 patients, 1.4 million controls) are still accounting for incomplete heritability; currently GWAS variants explain only from 1/4 to 1/3 of the total heritability of PD^16^. Another problem present in GWAS analyses is which are the functional and causal risk genes at each locus? At some loci the “nominated” genes at the GWAS signal are simply named for convenience while others are based on statistical inference. But, unlike familial mutations, risk genes remain largely unverified by direct confirmation. Moreover, we feel that the globally accurate but individually uncertain risk gene lists generated by computational methods are not good enough. When it comes to interpreting GWAS data, the field should not be satisfied with polygenic risk prediction or general biological inferences made without further mechanistic investigation. Ultimately, just as for familial PD gene mutations, if we cannot answer *where, when, and how* a variant functionally manifests, we do not understand the genetic risk specifically, and genetic architecture, generally. We (herein and ref. ^37^) and others^13^ have outlined strategies to investigate post-GWAS functionality. We advocate for more effort (and patience) to explore risk mechanisms in-depth, taking into account the complex nature of cell heterogeneity, chromatin regulation, and developmental stage. Unfortunately, not only do these approaches each have drawbacks, which would filter out a subset of risk, they do not by themselves get at disease origin. The sheer number of risk variants will likely require a great deal of separate experiments and many negative results. It almost goes without saying, that those negative results probably need accounting, which is not satisfied by current publication systems. Ultimately, the post-GWAS project may require large consortia to coordinate collaborative efforts between many labs, just as is required for the GWAS studies themselves^45^. Given a sufficient catalog of verified risk variant mechanisms, the hard task of linking those mechanisms directly to disease etiology can proceed. Perhaps only then will the full potential of GWAS utility will come to bare on complex diseases, such as PD.
